# A Prospective Study Assessing Wound Complications in a Series of Foot and Ankle Patients Treated With Enoxaparin: A Baseline for Future Studies

**DOI:** 10.7759/cureus.21167

**Published:** 2022-01-12

**Authors:** Andrew Walls, Brian Hanratty, Bakhat Yawar, Adam Tucker, Peter Stavrou, Sunil Ramawat, George Dracopoulos, Lukas Iselin

**Affiliations:** 1 Trauma and Orthopaedics, The Western Trust HSCNI, Derry/Londonderry, GBR; 2 Urology, The Western Trust HSCNI, Derry/Londonderry, GBR; 3 Trauma and Orthopaedics, Repatriation General Hospital, Adelaide, AUS; 4 Trauma and Orthopaedics, Blacktown Service Hospital, Blacktown, AUS; 5 Trauma and Orthopaedics, Burnside War Memorial Hospital, Adelaide, AUS; 6 Trauma and Orthopaedics, Luzerner Kantonsspital, Lucerne, CHE

**Keywords:** foot and ankle surgeon, low molecular weight heparin (lmwh), postoperative wound infection, surgical wound infection, delayed wound healing

## Abstract

Background and objective

Low-molecular-weight heparin (LMWH) prophylaxis has now become the gold-standard practice in patients requiring lower limb immobilization. We had noticed an increase in the incidence of wound-healing problems at our center, and the severity of the problems was found to be worse in patients undergoing foot and ankle surgery since we had adopted this practice. In this study, we aimed to describe the incidence and severity of wound-healing problems in this group of patients.

Methods

This was a prospective study and we collected data on the frequency and severity of wound problems occurring in patients undergoing a variety of foot and ankle operations. All patients underwent a standard agreed-on method of wound closure and dressings. Wounds were reviewed after two weeks and wound characteristics were noted using a rigid proforma. The primary outcome measure was to determine the incidence of delayed wound healing (DWH) and wound infections requiring antibiotics. Secondary outcomes were the characteristics of each delayed-healing wound.

Results

A total of 158 patients met the inclusion criteria of the study. One patient was not given postoperative LMWH and was excluded from the final analysis. Seven patients (4.5%) were noted to have DWH and four patients (2.6%) had a wound infection at the two-week postoperative follow-up. None of the patients required a second operation. Among patients with wound-healing problems, wound contour irregularities were noted in 51% and margin separation was noted in 65%.

Conclusion

The overall incidence of wound-healing problems such as DWH and wound infections was low in patients receiving prophylactic LMWH for foot and ankle surgery. Where postoperative wound problems did occur, these were associated with poor wound characteristics such as margin separation or contour irregularity. Further studies should be conducted to ascertain if the use of LMWH leads to problems with wound appearance.

## Introduction

Delayed wound healing (DWH) in foot and ankle surgery patients can lead to significant morbidity. Since the introduction of low-molecular-weight heparin (LMWH) prophylaxis guidelines [1], we had perceived a change in the frequency and characteristics of wound healing. LMWH for the prophylaxis of venous thromboembolism (VTE) has become standard practice in developed healthcare systems. Patients who require lower limb immobilization are particularly targeted for risk assessment, and there is increasing evidence from large systematic reviews that it can reduce the rate of deep venous thrombosis (DVT) in the outpatient setting [2]. In our facility, it is prescribed routinely to patients with lower limb immobilization following surgery or trauma. It was felt that the incidence of wound healing problems had increased; moreover, it was also observed that when a wound-healing problem occurred, its severity tended to be high. In this study, our objective was to describe and quantify wound-healing problems in consecutive patients undergoing a range of foot and ankle surgeries. We believe this study will serve as a base for future studies on wound healing in foot and ankle surgery.

The aim of this study was to prospectively measure the frequency of wound problems that were occurring in a consecutive cohort of patients who underwent foot and ankle surgery and describe the characteristics of each wound. Given the seriousness of thromboembolic events, a controlled trial was not possible or ethically viable. The primary outcomes were the incidence of DWH and wound infection requiring an antibiotic prescription. The secondary outcomes were the characteristic of each delayed-healing wound. A correlation analysis between individual procedures and patient comorbidities was also carried out.

## Materials and methods

A prospective observational study was performed between October 2013 and June 2014. All surgical procedures were carried out by two fellowship-trained foot and ankle surgeons across five different sites. This study was designed as an audit of clinical practice to ascertain the incidence and severity of wound complications in ankle surgery with the use of LMWH. The wound assessments were carried out at two sites by the senior authors. The sample size was determined based on the data from the first 30 patients. To attain a precision of 10%, it was determined that a sample size of 150 patients would be required, and this was approximately the proportion achieved ±5%. This precision calculation described precision as being exact upper 95% confidence interval (CI) minus exact lower 95% CI.

The inclusion criteria were as follows: consecutive patients undergoing foot and ankle surgery performed by senior surgeons and treated with the LMWH enoxaparin. The exclusion criteria were as follows: patients receiving anticoagulation other than enoxaparin, and children. A total of 158 suitable patients were identified, out of which 91 were male and 67 were female.

**Figure 1:**
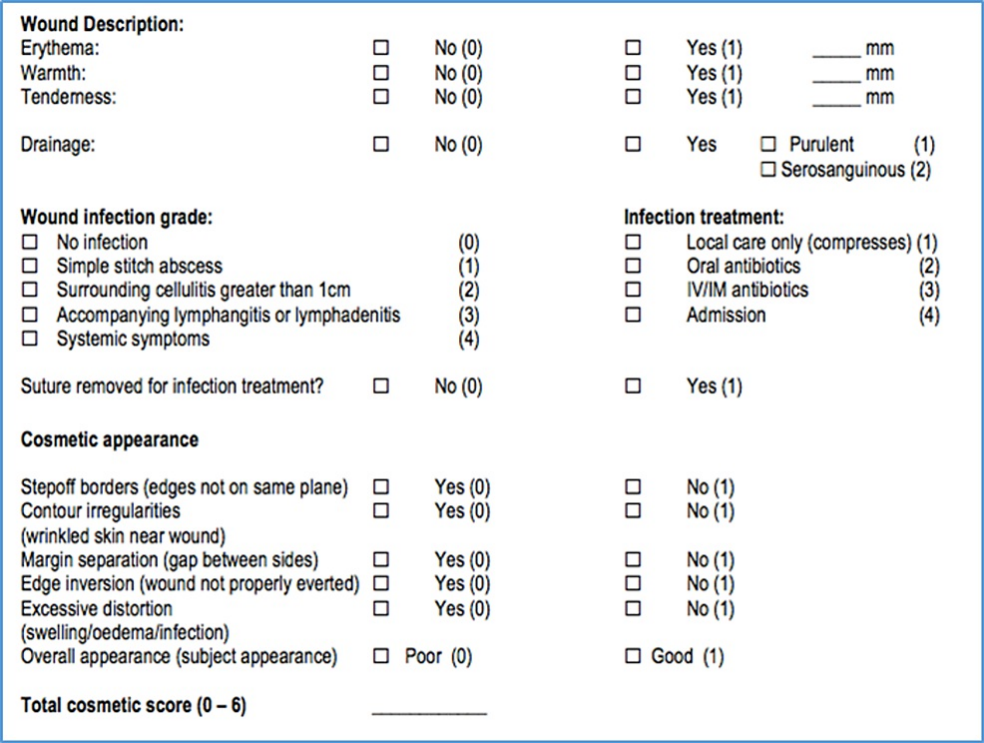
Rigid proforma for data collection regarding wounds IV: intravenous, IM: intramuscular

For all operations, standard wound closure was performed using a 2/0 Vicryl subcutaneous stitch, followed by a 3/0 Monocryl running mattress stitch to the skin. Nylon was only used at the discretion of the surgeon if there were concerns about the integrity of the skin upon closure. Wound dressings were standardized for all surgeries. Postoperatively, enoxaparin 40 mg (an LMWH) was prescribed once daily, with the first dose commencing on the first day postoperatively. A routine review was performed at two weeks postoperatively by the operating surgeon and the attending fellow. Wound assessments were carried out using a rigid proforma (Figure 1).

Each wound was described and allocated two scores. The first was a numerical “cosmetic score” that ranged from 0-6 with 6 being the best outcome and 0 being the worst. The second was a binary score, which was marked as either good or poor. Descriptive data described if sutures were required to be removed and if the wound had been kept clean, apart from documenting if there was any erythema, warmth, or tenderness. If a discharge was present, it was described as either serosanguinous, bloody, or purulent. A description of the wound included whether or not there were step-off borders, wrinkled irregularities, margin separation, edge eversion, and, lastly, excessive distortion. The Chi-square analysis was used to find the association between wound descriptors and incidence of infection.

## Results

A total of 158 patients met the inclusion criteria, of which 91 (57.6%) were males and 67 (42.4%) were females. The mean age of the cohort was 49.2 ±16.9 years (range: 15-76). One patient was lost to follow-up, leaving 157 for the final analysis. Comorbidities included three patients with diabetes and two with rheumatoid arthritis. Only nine patients admitted to being smokers, and three were ex-smokers. Patient comorbidities are listed in Table 1. Some patients had multiple comorbidities.

**Table 1:** Incidence of comorbidities in the patient group ASA: American Society of Anesthesiologists

Comorbidity	Number of patients
Diabetes	3
Rheumatoid arthritis	2
Smoking	9
ASA score >2	15

**Figure 2:**
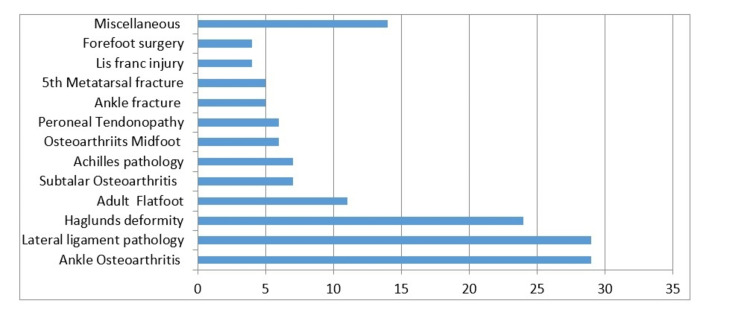
Range of pathologies included in the study

A wide range of foot and ankle pathology was captured in this study with over 22 different procedures performed. The details are laid out in Figure 2.

Cephalosporin antibiotic prophylaxis was provided to 155 patients (98.1%). One patient received a macrolide, one received aminoglycoside, and one received no prophylaxis. All but one patient received postoperative enoxaparin venous thromboprophylaxis at 40 mg OD. A dose of 80 mg OD was given to an obese rheumatoid patient undergoing a triple fusion [3].

Overall, 61% of procedures were performed in the morning. A lower limb pneumatic tourniquet was used in 145 procedures (91.8%), with a mean duration of 48.5 ±27.7 minutes (range: 10-177). A pressure of 250 mmHg was used in 101/145 (70.0%) procedures, with the remainder performed at a pressure of 300 mmHg. There was no correlation between the presence of a postoperative wound infection and tourniquet time, nor inflation pressure.

Follow-up was performed at a mean of 14.7 ±4.9 days. The Median follow-up was 14 days postoperatively. At this time, seven (4.5%) wounds had erythema, six (3.8%) demonstrated warmth, and seven (4.5%) were tender to palpate. One patient had purulent discharge, four had serosanguinous discharge, and two had frank blood leakage from the wound. All of these wounds were managed with simple wound dressings and close observation.

Four patients (2.5%) had cellulitis of >1 square cm area, one ankle arthroscopy and ligament reconstruction, one calcaneal open reduction and internal fixation (ORIF), one lesser metatarsal heads excision, and one hallux valgus correction. All of these patients were receiving 40-mg prophylactic enoxaparin. One had no tourniquet applied (metatarsal resection), two had a tourniquet pressure of 250 mmHg both for less than one hour of surgical time, and one had a tourniquet pressure of 300 mmHg. None of those who developed cellulitis had significant comorbidity, nor did anyone demonstrate any associated lymphangitis requiring systemic therapy or hospital admission. All were successfully treated with oral antibiotic therapy as an outpatient. Wound assessments were performed, which are summarized in Table 2.

**Table 2:** Incidence of the type of wound abnormalities noted at clinical follow-up

	Step-off	Irregular	Separation	Inverted	Distorted	Good appearance
Present, n (%)	4 (3%)	15 (10%)	20 (13%)	3 (2%)	5 (3%)	148 (94%)
Absent, n (%)	153 (97%)	142 (90%)	137 (87%)	154 (98%)	151 (96%)	7 (4%)
Not recorded, n (%)	0	0	0	0	1 (1%)	2 (2%)

Overall, the cosmetic score ranged from 1 to 6 (mean: 5.6, median: 6). Chi-Square analysis for each cosmetic criteria was performed. The presence of separated edges, a distorted wound, or a poor appearance demonstrated a statistically significant association with a postoperative wound problem, e.g., cellulitis (Table 3).

**Table 3:** Association between the appearance of wound and risk of infection (Chi-square analysis) *Statistically significant

Wound descriptor	Association with wound infection	
	X^2^	P-value
Step-off	11.258	0.077
Irregular	1.894	0.269
Separation	22.783	0.001*
Inverted	15.556	0.058
Distorted	89.976	<0.001*
Good appearance	62.965	<0.001*

Wound grades were numerically coded against the total cosmetic score. The overall total cosmetic score was inversely related to wound grades (r = -0.419, p<0.01).

DWH was observed in 4.5% of foot and ankle surgery patients who were prescribed enoxaparin. No patient was required to return to the theater, while 2.6% suffered from wound infection. There was a wide range of characteristics; contour irregularities (wrinkled skin near the wound) were described in 51% of the wounds that had problems. Margin separation was described in 65%.

## Discussion

The Cochrane database has concluded that the use of LMWH significantly reduces VTE when immobilization of the lower leg is required. This systematic review by Testroote et al. included over 1,490 patients who had a lower limb injury that had been immobilized in a plaster cast or a brace for at least one week, and who received no prophylaxis or placebo [4]. They found an incidence of VTE ranging from 4.3% to 40%. This number was significantly lower in patients who received daily subcutaneous injections of LMWH. Complications of major bleeding events were extremely rare (0.3%), and there were no reports of heparin-induced thrombocytopenia.

Wukich et al. prospectively evaluated 1,465 consecutive foot and ankle surgical cases performed by a single surgeon [5]. The overall surgical site infection (SSI) rate in this study was 3.5%, with significantly more infections occurring in individuals with diabetes than in those without (9.5 vs. 2.4%, p<0.001). Peripheral neuropathy, Charcot neuroarthropathy, current or past smoking, and longer duration of surgery were significantly associated with SSI on multivariate analysis. Other studies have confirmed that postoperative infections occur significantly more in diabetic patients compared to non-diabetics (13.2 vs. 2.8%) [6]. Interestingly, the three diabetic patients in our study did not have wound problems. With regard to the two rheumatoid patients, one had grade 5 margin separation and contour irregularities. He underwent a hindfoot fusion and was taking clopidogrel. The other patient had no wound complications. Our study recorded a lower SSI rate than Wukich et al. at 2.6%. However, the overall rate of delayed healing was 4.5%.

Kadota et al. reviewed the risk factors for SSI and DWH after orthopedic surgery in rheumatoid arthritis patients [7]; 1,036 patient records were reviewed and logistic regression analysis was carried out based on age, body mass index, disease duration, preoperative laboratory data, surgical procedure, corticosteroid use, comorbidity, and the use of conventional synthetic disease-modifying antirheumatic drugs (DMARDS) and biological DMARDS as variables. SSI and DWH were identified in 19 (1.8%) and 15 (1.4%) cases respectively. Foot and ankle surgery was associated with an increased risk of SSI [odds ratio (OR): 3.167]. They concluded that foot and ankle surgery, total knee arthroplasty, and longer disease duration were risk factors for SSI and DWH.

Of the nine smokers, three had delayed healing (two 5s and one 4). Contour irregularity and margin separation were described in two of these wounds. Among the patients with comorbidities, five had poor scores. The conditions included chronic renal failure, hepatitis C, ischaemic heart disease, factor V Leiden deficiency, and unknown congenital syndrome.

The initial concern that enoxaparin was contributing to the incidence of wound-healing problems was not validated as per the findings in our study. In fact, the incidence of wound-healing problems in our cohort of foot and ankle surgery patients who were prescribed enoxaparin was found to be low. This study can reassure other surgeons that enoxaparin is safe to use in clinical practice. Regarding the characteristics of wounds that had healing issues, this study would indicate that those with an irregular border and separation represent more of a concern. Future studies comparing wound appearance with other prophylaxis are required to confirm if this is specific to enoxaparin. The wound descriptor table we have used in our study to grade wounds and their cosmetic appearance, albeit not used in previous literature, can be used in future studies to provide more evidence for its validity in larger cohorts of patients.

## Conclusions

In our study, DWH was observed in 4.5% of foot and ankle surgery patients who were prescribed enoxaparin. No patient was required to return to the theater, while 2.6% suffered wound infection. All infections were managed with oral antibiotics. The wounds were characterized by an increased margin of separation and contour irregularities. Whether enoxaparin contributes to the wound appearance is unclear. The findings of this study can be used in future comparative studies.
